# Assessment of the efficacy of various neoadjuvant anti-HER2 targeted therapies combined with chemotherapy for HER2-positive breast cancer in the real-world setting and development of a predictive model for pathological complete response

**DOI:** 10.3389/fonc.2025.1673810

**Published:** 2025-11-17

**Authors:** Haoqi Wang, Shan Gao, Zihao Bai, Lijia Wang, Cuizhi Geng

**Affiliations:** 1 Department of Breast Center, The Fourth Hospital of Hebei Medical University, Shijiazhuang, Hebei, China; 2 Hebei Key Laboratory of Breast Cancer Molecular Medicine, The Fourth Hospital of Hebei Medical University, Shijiazhuang, Hebei, China; 3 Department of Gland Surgery, The Hebei Province People’s Hospital, Shijiazhuang, Hebei, China; 4 Department of Computed Tomography and Magnetic Resonance, The Fourth Hospital of Hebei Medical University, Shijiazhuang, Hebei, China

**Keywords:** real world, HER2-positive breast cancer, neoadjuvant therapy, different anti-HER2 targeted therapies, pathological complete response, nomogram

## Abstract

**Background:**

The development of a robust and clinically applicable predictive model for pathological complete response (pCR) following neoadjuvant therapy (NAT) in human epidermal growth factor receptor 2 (HER2)-positive breast cancer (BC) is of critical importance.

**Methods:**

In this retrospective study, 393 female patients with stage II–III BC who received NAT followed by surgery between May 2021 and December 2023 were included. Clinicopathological data, apparent diffusion coefficient (ADC) values from breast MRI, and pathological remission after NAT were collected. The change rate of ADC values after two cycles of NAT (ΔADC_0-2_%) was calculated. The efficacy of NAT regimens containing trastuzumab plus pertuzumab (HP) and trastuzumab plus pyrotinib (HPy) was compared. A nomogram predicting pCR was constructed, and its performance was evaluated. The model was internally validated using the bootstrap resampling method.

**Results:**

The rate of total pathological complete response (tpCR) in the overall population was 68%. There was no statistically significant difference in tpCR between the HP and HPy groups (*P* > 0.05). Hormone receptor (HR) negativity, HER2 3+, high Ki-67 index, moderate-highly (M-H) infiltrated tumor-infiltrating lymphocytes (TILs), and ΔADC_0-2_% > 36.2% were independently associated with tpCR (*P* < 0.05). The nomogram integrating these variables exhibited good discrimination (AUC, 0.75) and calibration ability (*P* = 0.925), as well as valuable clinical applicability.

**Conclusion:**

Both HP and HPy combined with chemotherapy can be considered as optional NAT regimens for HER2-positive BC. The nomogram incorporating common clinical indicators provides a basis for clinicians to predict NAT efficacy at an earlier stage.

## Introduction

Human epidermal growth factor receptor 2 (HER2)-positive breast cancer is a subtype of breast cancer (BC), characterized by HER2 amplification and accounting for 20-25% of all BC cases (1). Neoadjuvant therapy (NAT) is a crucial preoperative systemic therapy for HER2-positive BC, facilitating tumor downstaging to render it operable and breast-conserving (2). It also assesses drug sensitivity, thereby optimizing postoperative adjuvant treatment plans (3). According to the NeoSphere (4) and PEONY (5) clinical trials, chemotherapy combined with trastuzumab (H) and pertuzumab (P) is the standard of care for HER2-positive BC in NAT. Based on the PHEDRA study (6), pyrotinib (Py), a small molecule tyrosine kinase inhibitor originally developed in China, in combination with H and docetaxel, has also become an optional NAT regimen for patients with HER2-positive tumors. Although the combination of two different anti-HER2 targeted drugs significantly improves pathological response compared to single-targeted treatment with H, there are currently no randomized controlled clinical trials comparing the two combination regimens.

Pathological complete response (pCR) is a crucial indicator for assessing the effectiveness of NAT (7). Patients with HER2-positive tumors who achieve pCR through NAT tend to have significantly prolonged survival (7). However, pCR can only be confirmed through pathological testing of the tumor bed after surgery. If the efficacy of NAT could be predicted earlier, allowing for timely adjustment of the therapeutic regimen, the likelihood of achieving pCR and improving prognosis would be significantly enhanced. To date, numerous clinicopathological indicators and even multidimensional radiomics have been incorporated into predictive models for pCR in order to improve the accuracy of predictions (8–12). However, there is a scarcity of models specifically designed for the HER2-positive subtype, and the parameters included in existing models are often complex and not readily accessible (8–12), which limits their widespread application by clinicians. Therefore, it is essential to construct a predictive model specifically for HER2-positive breast cancer that incorporates routine and readily available clinicopathological and imaging parameters, making it more practical for clinical use.

Owing to its superior soft tissue resolution and multiparametric imaging capabilities, MRI is considered the most accurate imaging modality for evaluating the efficacy of NAT (13–16). Both imaging and clinical guidelines recommend MRI for this purpose. The apparent diffusion coefficient (ADC) is the most commonly used parameter in MRI diffusion-weighted imaging (DWI). It describes the speed and range of molecular diffusion in different directions of the DWI sequence, reflecting the random motion of water molecules within tissue. High ADC values typically indicate free movement of water molecules, while low ADC values suggest restricted movement, which may be associated with high cellular density. Thus, ADC values can not only distinguish between benign and malignant tumors (17, 18), but also provide valuable reference for assessing the efficacy of NAT (19, 20). During NAT, if the treatment is effective, the ADC value will increase as cancer cell density decreases (17, 21–23). Numerous studies have confirmed that ADC values and their changes are closely related to NAT efficacy (12, 24). Moreover, it has been proposed that early changes in ADC values can better predict pCR after NAT. Clinically, ADC values are routinely recorded in standard MRI reports, offering valuable insights for clinicians to evaluate patients’ conditions.

Consequently, this study was designed to compare the efficacy of NAT regimens containing HP (trastuzumab plus pertuzumab) and HPy (trastuzumab plus pyrotinib) in a real-world setting. It also aimed to explore the correlation between the early change rate of the ADC value (after two cycles of NAT) and the efficacy of NAT for HER2-positive BC. Univariate and multivariate analyses were conducted to identify predictors of pCR and to construct a predictive nomogram that could forecast the probability of pCR at an earlier stage.

## Materials and methods

### Patients

Patients who received NAT at the Fourth Hospital of Hebei Medical University between May 2021 and December 2023 were included in this study. The inclusion criteria were as follows: (1) Female, (2) Pathologically confirmed HER2-positive primary BC, (3) No prior treatment before NAT, (4) Completion of the full course of NAT followed by surgery, (5) Periodic breast contrast-enhanced (CE)-MRI examinations (before and after NAT, and every two cycles during NAT), (6) Availability of complete clinicopathological information and imaging data. The exclusion criteria were: (1) Bilateral or occult breast cancer, (2) Incomplete NAT or surgery, (3) Insufficient clinicopathological data, (4) Loss to follow-up. The study was approved by the Ethics Committee of the Fourth Hospital of Hebei Medical University, in accordance with the Helsinki Declaration of 1975.

### Therapeutic regimens

Chemotherapy combined with dual anti-HER2 drugs is the standard of care for HER2-positive BC in NAT. The chemotherapy regimens used included:TCb: Albumin-bound paclitaxel (T, 250 mg/m²) plus carboplatin (Cb, AUC = 6), administered for six cycles. AC-T: Pirarubicin (A, 60 mg/m²) or doxorubicin liposome (35 mg/m²) or epirubicin (90 mg/m²) plus cyclophosphamide (C, 600 mg/m²) for four cycles, followed by albumin-bound paclitaxel (T, 250 mg/m²) for four cycles. TA: Albumin-bound paclitaxel (T, 250 mg/m²) plus pirarubicin (A, 50 mg/m²) or doxorubicin liposome (35 mg/m²) or epirubicin (75 mg/m²), administered for six cycles. T: Albumin-bound paclitaxel (T, 250 mg/m²) alone, administered for six cycles. The anti-HER2 targeted combinations included: HP: Trastuzumab (H, loading dose 8 mg/kg, maintenance dose 6 mg/kg) plus pertuzumab (P, loading dose 840 mg, maintenance dose 420 mg). HPy: Trastuzumab (H, loading dose 8 mg/kg, maintenance dose 6 mg/kg) plus pyrotinib (Py, initial dose 400 mg, with dose reduction to 320 mg or even 240 mg based on adverse events (AEs), taken orally once daily). These combinations were administered concurrently with chemotherapy throughout NAT. All regimens, except for pyrotinib, were administered intravenously on day 1 every 21 days. Dose reductions or delays were permitted for chemotherapy and pyrotinib based on AEs. Dose reductions were not allowed for trastuzumab and pertuzumab.

### Clinicopathologic data collection and definitions

The clinicopathological data collected included age, menstrual status, T stage, axillary lymph node metastasis and N stage, TNM stage (AJCC version 8.0), hormone receptor (HR) status, HER2 expression, Ki-67 index, tumor-infiltrating lymphocytes (TILs), the change rate of the apparent diffusion coefficient (ΔADC_0-2_%), NAT regimens, surgical method, Miller-Payne (MP) grading, and residual cancer burden (RCB) classification. The estrogen receptor (ER), progesterone receptor (PR), HER2, and Ki-67 were evaluated using immunohistochemical (IHC) staining.

HR-positive status was defined as ER and/or PR expression of ≥1%, while HR-negative status was assigned to cases with expression levels below this threshold (25). HER2-positive status was determined by IHC staining showing 3+ or 2+ with confirmatory fluorescence *in situ* hybridization (FISH) positivity. TILs were assessed via hematoxylin-eosin (HE) staining and categorized as low (L, 0%-10%), moderate (M, 11%-59%), and high (H, >60%) (26). ΔADC_0-2_% was calculated as (ADC value after two cycles of NAT − ADC value pre-NAT)/ADC value pre-NAT×100%. Total pathological complete response (tpCR) was defined as the absence of residual invasive cancer cells in both the breast and lymph nodes (ypT0/is and ypN0), corresponding to residual cancer burden (RCB) 0. Breast pathological complete response (bpCR) was defined as the absence of residual invasive cancer in the breast, equivalent to Miller-Payne (MP) grade 5.

### Statistical analysis

Data analysis was performed using SPSS version 27.0, R software (version 4.0), and MedCalc 20.0. Continuous variables were expressed as mean ± standard deviation (SD) or median (interquartile range), and intergroup comparisons were made using the t-test or nonparametric tests as appropriate. Categorical variables were presented as frequencies with percentages, and differences between groups were assessed using the Chi-square test or Fisher’s exact test. Propensity score matching (PSM) was conducted at a 1:2 ratio to adjust for confounding variables between the HP and HPy groups using R software. Multivariate binary logistic regression analysis was performed to identify independent predictors of pCR. A predictive nomogram for pCR was developed using the ‘rms’ package in R software. Receiver operating characteristic (ROC) curves were generated using the ‘pROC’ and ‘ggplot2’ packages to calculate the area under the curve (AUC) and determine the optimal cutoff values, specificity, and sensitivity. ROC curves comparing each variable and the nomogram were plotted using MedCalc 20.0 software, and the corresponding AUC, optimal cutoff values, specificity, and sensitivity were calculated. The calibration accuracy of the model was assessed using the Hosmer-Lemeshow Calibration Curve. Decision curve analysis (DCA) was performed using the ‘rmda’ package to evaluate the clinical utility of the nomogram. Internal validation of the model was conducted using the Bootstrap resampling method. A two-tailed *P* value of less than 0.05 was considered statistically significant.

## Results

### Patient characteristics

**Figure 1:**
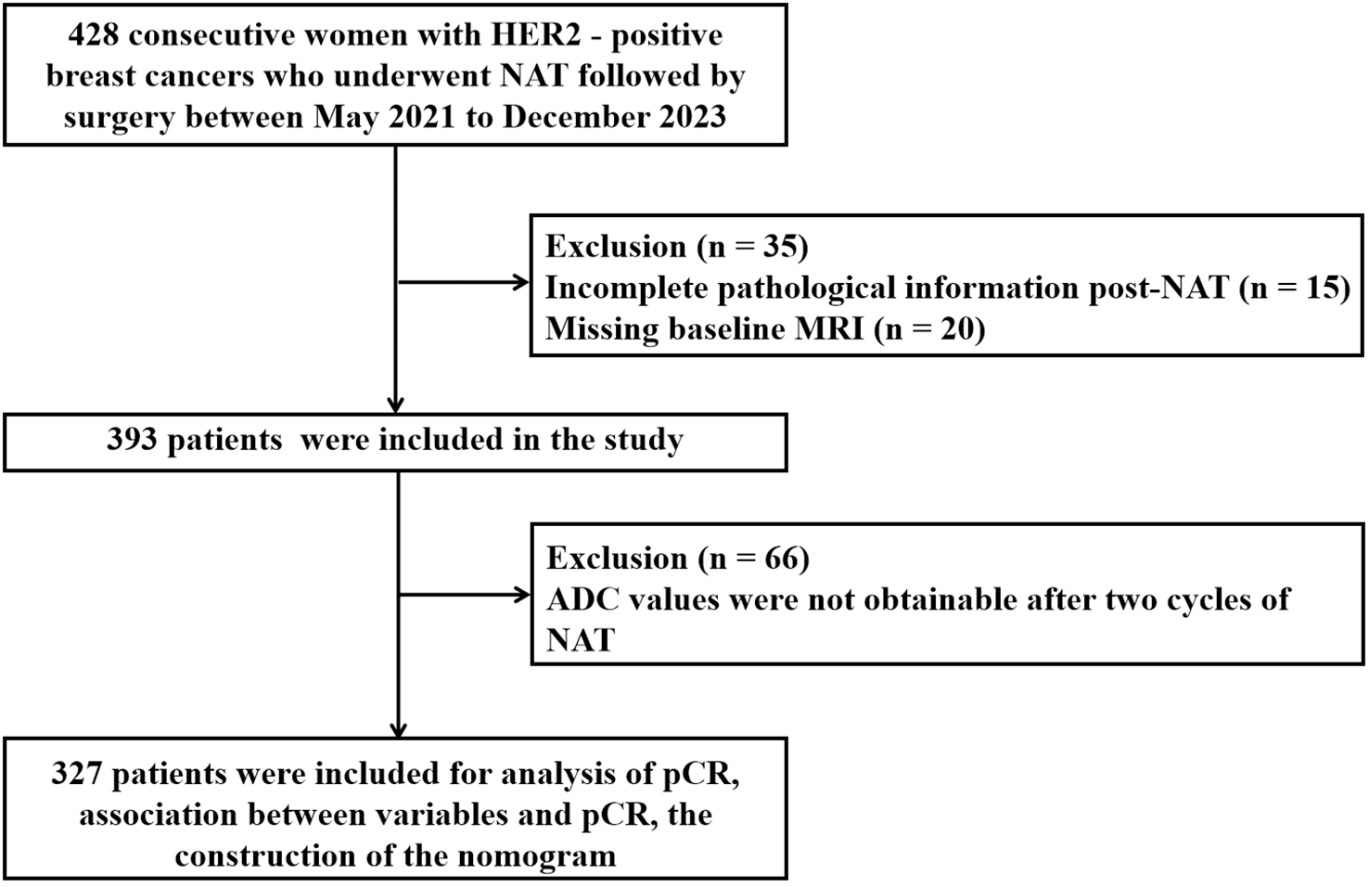
Flowchart shows patient exclusion for the study.

Between May 2021 and December 2023, a total of 428 patients diagnosed with HER2-positive BC underwent NAT. Of these, 35 patients were excluded due to incomplete pathological information post-NAT (n = 15) and missing baseline MRI (n = 20) (**Figure 1**). Consequently, 393 patients (median age, 51 years) were included in the study. At initial diagnosis, 57% of the patients were premenopausal. The majority of patients were staged as T2 (67%), N1 (62%), and stage II (62%). The predominant pathological histological type was invasive ductal carcinoma (83%), with most histological grades ranging from 1 to 2 (86%). The proportion of HR negativity was 44%, while the positive rate was 56%. HER2 amplification was primarily manifested as 3+ (92%). A relatively high percentage of patients (55%) exhibited high expression of Ki-67 (> 30%). TILs predominantly showed low infiltration (60%). The most commonly used chemotherapy regimen was TCb*6 (57%), followed by T*6 (23%) and AC*4 - T*4/TA*6 (20%). The primary anti-HER2 targeted combination was HP (77%) (**Table 1**).

**Table 1:** Characteristics of included patients.

Characteristics	Included patients (n = 393)
Age, n(%)	
≤50	177 (45)
>50	216 (55)
Menstrual Status, n(%)	
Premenopause	224 (57)
Postmenopause	169 (43)
Pre-NAT ^a^ Clinical T Stage, n(%)	
cT1	63 (16)
cT2	263 (67)
cT3	38 (10)
cT4	29 (7)
Pre-NAT^a^ Clinical N Stage, n(%)	
cN0	36 (9)
cN1	243 (62)
cN2	13 (3)
cN3	101 (26)
Pre-NAT^a^ Clinical TNM Stage, n(%)	
II	242 (62)
III	151 (38)
Pre-NAT^a^ Histopathological Type, n(%)	
Invasive ductal carcinoma	328 (83)
others	65 (17)
Pre-NAT^a^ Histological Grade, n(%)	
G1-2	339 (86)
G3	54 (14)
Pre-NAT^a^ HR^b^ State, n(%)	
Negative	172 (44)
Positive	221 (56)
Pre-NAT^a^ HER2 Expression, n(%)	
2 +	32 (8)
3 +	361 (92)
Pre-NAT^a^ Ki-67 Expression, n(%)	
≤30%	175 (45)
>30%	218 (55)
Pre-NAT^a^ TILs^c^ Infiltration, n(%)	
Low infiltrated	234 (60)
M-H^d^ infiltrated	159 (40)
△ADC_0-2_%^e^, n(%)	
≤ 36.2%	188 (48)
> 36.2%	139 (35)
missing	66 (17)
Chemotherapy Regimen for NAT, n(%)	
platinum contained	224 (57)
anthracyclines contained	80 (20)
monoalbumin-bound paclitaxel	89 (23)
Anti-HER2 Regimen for NAT^a^, n(%)	
HP	303 (77)
HPy	90 (23)
Surgery of Breast post-NAT^a^, n(%)	
Mastectomy	313 (80)
Breast-conserving surgery	80 (20)
Surgery of Axilla post-NAT^a^, n(%)	
Sentinel lymph node biopsy	27 (7)
Axillary lymph node dissection	366 (93)

**a** NAT: neoadjuvant therapy, **b** HR: hormone receptor, **c** TILs: tumor-infiltrating lymphocytes, **d** M-H: moderate-high, **e** △ADC_0-2_%: change rate of ADC after two cycles of neoadjuvant therapy.

### Pathological response after NAT of the overall population and the optimal cutoff of ΔADC_0–2_% for tpCR

**Figure 2:**
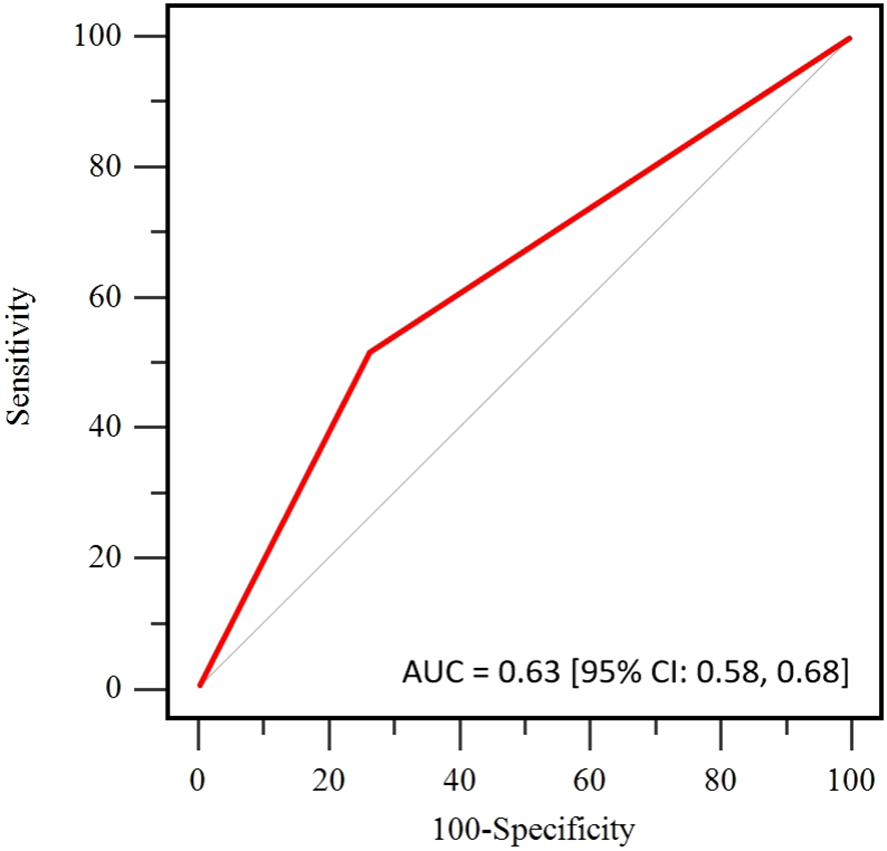
Receiver operating curve (ROC) of the apparent diffusion coefficient (ADC) for pathological complete response (pCR) prediction.

All patients underwent surgery within 2 to 4 weeks after completing NAT, with 80% undergoing mastectomy and 20% undergoing breast-conserving surgery. The tpCR rate for the entire cohort was 68%, and the pathological complete response rate in the breast (bpCR) was 76%. Due to significant tumor regression, ADC values were not obtainable for 66 patients after two cycles of NAT. For the remaining patients with available ΔADC_0-2_%, the tpCR rate was 64%, and the bpCR rate was 71%. According to the ROC curve for ΔADC_0-2_% (**Figure 2**), the AUC was 0.63 [95% CI: 0.58, 0.68], with an optimal cutoff value of 36.2% for predicting tpCR. Based on this cutoff, patients were divided into two groups for further analysis: those with ΔADC_0-2_% ≤ 36.2% and those with ΔADC_0-2_% > 36.2%.

### Comparison of demographic characteristics and pathological response between the HP group and the HPy group

#### Before PSM

Before PSM (**Table 2**), statistically significant differences were observed between the two groups in terms of patient age, menstrual status, histological type, and the choice of chemotherapy regimen (*P* < 0.05). Compared with patients treated with HP, a higher proportion of patients in the HPy group were over 50 years old (64%), postmenopausal (52%), had a histological type of non-invasive ductal carcinoma (26%), and received a chemotherapy regimen including anthracyclines (51%). No significant differences were found between the two groups in other clinical and pathological indicators (*P* > 0.05). The tpCR rate was numerically higher in the HP group than in the HPy group, but the difference was not statistically significant (69% vs 64%, *P* = 0.419). The bpCR rate was significantly higher in the HP group (79% vs 67%, *P* = 0.061).

**Table 2:** Comparison of clinicopathological characteristics between the HP and HPy groups before PSM.

Characteristics	HP (n=303)	HPy (n=90)	*P* Value
Age, n(%)			0.039
≤ 50	145 (48)	32 (36)	
> 50	158 (52)	58 (64)	
Menstrual Status, n(%)			0.044
premenopause	181 (60)	43 (48)	
postmenopause	122 (40)	47 (52)	
Pre-NAT^a^ Clinical T Stage, n(%)			0.701
cT1	47 (16)	16 (18)	
cT2	207 (68)	56 (62)	
cT3	27 (9)	11 (12)	
cT4	22 (7)	7 (8)	
Pre-NAT^a^ Clinical N Stage, n(%)			0.469
cN0	29 (10)	7 (8)	
cN1	181 (60)	62 (69)	
cN2	10 (3)	3 (3)	
cN3	83 (27)	18 (20)	
Pre-NAT^a^ Clinical TNM Stage, n(%)			0.524
II	186 (61)	59 (66)	
III	117 (39)	31 (34)	
Pre-NAT^a^ Histopathological Type, n(%)			0.009
Invasive ductal carcinoma	261 (86)	67(74)	
others	42 (14)	23 (26)	
Pre-NAT^a^ Histological Grade, n(%)			0.409
G1-2	259 (85)	80 (89)	
G3	44 (15)	10 (11)	
Pre-NAT^a^ HR^b^ State, n(%)			0.288
negative	137 (45)	35 (39)	
positive	166 (55)	55 (61)	
Pre-NAT^a^ HER2 Expression, n(%)			0.463
2+	23 (8)	9 (10)	
3+	280 (92)	81(90)	
Pre-NAT^a^ Ki-67 Expression, n(%)			0.325
≤ 30%	139 (46)	36 (40)	
> 30%	164 (54)	54 (60)	
Pre-NAT^a^ TILs^c^, n(%)			0.117
Low infiltrated	174 (57)	60 (67)	
M-H^d^ infiltrated	129 (43)	30 (33)	
ΔADC_0-2_%^e^, n(%)			0.167
≤ 36.2%	148 (49)	40 (45)	
> 36.2%	110 (36)	29 (32)	
missing	45 (15)	21 (23)	
Chemotherapy Regimen for NAT^a^, n(%)			<0.001
anthracyclines contained	34 (11)	46 (51)	
platinum contained	210 (69)	14 (16)	
monoalbumin-bound paclitaxel	59 (20)	30 (33)	
Surgery of Breast post-NAT^a^, n(%)			0.090
Mastectomy	247	66 (73)	
Breast-conserving surgery	56	24 (27)	
Surgery of Axilla post-NAT^a^, n(%)			0.953
Sentinel lymph node biopsy	21	6 (7)	
Axillary lymph node dissection	282	84 (93)	

**a** NAT: neoadjuvant therapy, **b** HR: hormone receptor, **c** TILs: tumor-infiltrating lymphocytes, **d** M-H: moderate-high, **e** △ADC_0-2_%: change rate of ADC after two cycles of neoadjuvant therapy.

#### After PSM

**Figure 3:**
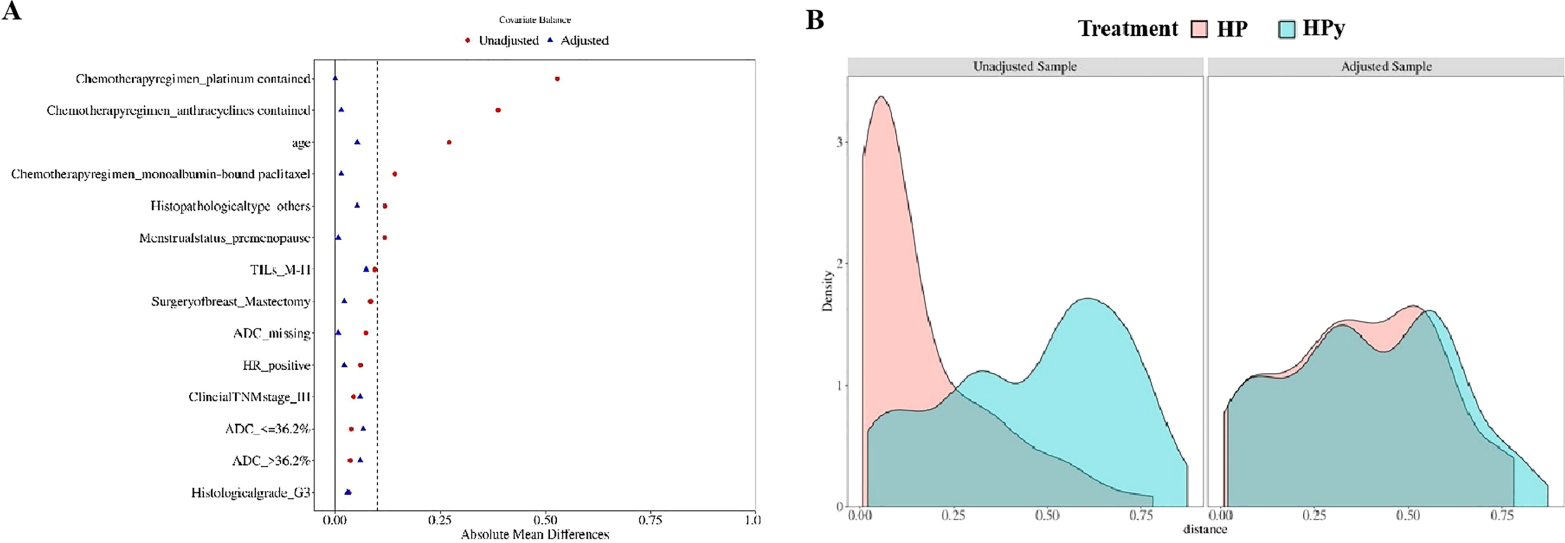
Testing the balance of covariates between the HP group and the HPy group before and after PSM. **(A)** Propensity score scatter plot before and after the PSM. **(B)** Distribution histogram of standard deviation.

To eliminate confounding factors, this study employed a 1:2 nearest neighbor matching without replacement based on propensity scores, with a caliper width set at 0.2. The post-matching analysis demonstrated a more uniform distribution of propensity scores between the two groups (**Figure 3A**), with the standard deviation clustering around zero (**Figure 3B**). Following PSM, the HP group included 106 individuals, and the HPy group included 67 patients. Tumor characteristics were well-balanced between the two groups (**Table 3**). After PSM, there was no statistically significant difference in the tpCR (65% vs 64%, *P* = 0.902) and bpCR (75% vs 66%, *P* = 0.211) rates between the HP and HPy groups.

**Table 3:** Comparison of clinicopathological characteristics between the HP and HPy groups after PSM.

Characteristics	HP (n=106)	HPy (n=67)	*P* Value
Age, n(%)			0.915
≤ 50	42 (40)	26 (39)	
> 50	64 (60)	41 (61)	
Menstrual Status, n(%)			0.980
premenopause	54 (51)	34 (51)	
postmenopause	52 (49)	33 (49)	
Pre-NAT^a^ Clinical T Stage, n(%)			0.133
cT1	20 (19)	8 (12)	
cT2	73 (69)	42 (63)	
cT3	7 (7)	10 (15)	
cT4	6 (5)	7 (10)	
Pre-NAT^a^ Clinical N Stage, n(%)			0.498
cN0	8 (8)	7 (11)	
cN1	64 (60)	42 (63)	
cN2	2 (2)	3 (4)	
cN3	32 (30)	15 (22)	
Pre-NAT^a^ Clinical TNM Stage, n(%)			0.474
II	69 (65)	40 (60)	
III	37 (35)	27 (40)	
Pre-NAT^a^ Histopathological Type, n(%)			0.274
Invasive ductal carcinoma	92 (87)	54 (81)	
others	14 (13)	13 (19)	
Pre-NAT^a^ Histological Grade, n(%)			0.308
G1-2	91 (86)	61 (91)	
G3	15 (14)	6 (9)	
Pre-NAT^a^ HR^b^ State, n(%)			0.835
negative	46 (43)	28 (42)	
positive	60 (57)	39 (58)	
Pre-NAT^a^ HER2 Expression, n(%)			0.916
2 +	9 (8)	6 (9)	
3 +	97 (92)	61 (91)	
Pre-NAT^a^ Ki-67 Expression, n(%)			0.873
≤ 30%	43 (41)	28 (42)	
> 30%	63 (59)	39 (58)	
Pre-NAT^a^ TILs^c^, n(%)			0.270
Low infiltrated	64 (60)	46 (69)	
M-H^d^ infiltrated	42 (40)	21 (31)	
ΔADC_0-2_%^e^, n(%)			0.429
≤ 36.2%	55 (52)	28 (42)	
> 36.2%	32 (30)	25 (37)	
missing	19 (18)	14 (21)	
Chemotherapy Regimen for NAT, n(%)			0.474
anthracyclines contained	35 (33)	28 (42)	
platinum contained	28 (26)	14 (21)	
monoalbumin-bound paclitaxel	43 (41)	25 (37)	
Surgery of Breast post-NAT^a^, n(%)			0.844
Mastectomy	89 (84)	57 (85)	
Breast-conserving surgery	17 (16)	10 (15)	
Surgery of Axilla post-NAT^a^, n(%)			0.298
Sentinel lymph node biopsy	3 (3)	5 (7)	
Axillary lymph node dissection	103 (97)	62 (93)	

**a** NAT: neoadjuvant therapy, **b** HR: hormone receptor, **c** TILs: tumor-infiltrating lymphocytes, **d** M-H: moderate-high, **e** △ADC_0-2_%: change rate of ADC after two cycles of neoadjuvant therapy.

### Clinicopathological indicators influencing tpCR

The analysis was conducted after excluding 66 patients who did not have ADC values after the second cycle of NAT. Among the clinicopathological and imaging characteristics (**Table 4**), positive lymph nodes (*P* = 0.015), HR negativity (*P* < 0.001), high Ki-67 index (*P* = 0.005), moderate-to-high (M-H) infiltrated TILs (*P* < 0.001), ΔADC_0-2_% > 36.2% (*P* < 0.001), HER2 3+ (*P* < 0.001), and the TCb regimen (*P* = 0.003) were associated with tpCR.

**Table 4:** Comparison of clinicopathological characteristics between the pCR and non-pCR groups in the overall population.

	Pathological response after NAT^a^		
Characteristics	non-pCR (n=118)	pCR (n=209)	*P* Value
Age, n(%)			0.263
≤50	50 (42)	102 (49)	
>50	68 (58)	107 (51)	
Menstrual Status, n(%)			0.259
Premenopause	63 (53)	125 (60)	
Postmenopause	55 (47)	84 (40)	
Pre-NAT^a^ Clinical T Stage, n(%)			0.72
cT1	14 (12)	33 (16)	
cT2	83 (71)	138 (66)	
cT3	10 (8)	21 (10)	
cT4	11 (9)	17 (8)	
Pre-NAT^a^ Clinical N Stage, n(%)			0.015
Negative	5 (4)	24 (11)	
Positive	113 (96)	185 (89)	
Pre-NAT^a^ Clinical TNM Stage, n(%)			0.816
II	73 (62)	132 (63)	
III	45 (38)	77 (37)	
Pre-NAT^a^ Histopathological Type, n(%)			0.96
Invasive ductal carcinoma	98 (83)	174 (83)	
others	20 (17)	35 (17)	
Pre-NAT^a^ Histological Grade, n(%)			0.111
G1-2	97 (82)	185 (89)	
G3	21 (18)	24 (11)	
Pre-NAT^a^ HR^b^ State, n(%)			<0.001
Negative	36 (31)	104 (50)	
Positive	82 (69)	105 (50)	
Pre-NAT^a^ HER2 Expression, n(%)			<0.001
2 +	20 (17)	9 (4)	
3 +	98 (83)	200 (96)	
Pre-NAT^a^ ki-67 Expression, n(%)			0.005
≤ 30%	66 (56)	83 (40)	
> 30%	52 (44)	126 (60)	
Pre-NAT^a^ TILs^c^ Infiltration, n(%)			<0.001
Low infiltrated	89 (75)	116 (55)	
M-H^d^ infiltrated	29 (25)	93 (45)	
ΔADC_0-2_%^e^, n(%)			<0.001
≤ 36.2%	87 (74)	101 (48)	
> 36.2%	31 (26)	108 (52)	
Chemotherapy Regimen for NAT^a^, n(%)			0.003
anthracyclines contained	27 (23)	43 (21)	
platinum contained	52 (44)	128 (61)	
monoalbumin-bound paclitaxel	39 (33)	38 (18)	
Anti-HER2 Regimen for NAT^a^, n(%)			0.085
HP	87 (74)	171 (82)	
HPy	31 (26)	38 (18)	
Surgery of Breast post-NAT^a^, n(%)			0.78
Mastectomy	98 (83)	171 (82)	
Breast-conserving surgery	20 (17)	38 (18)	
Surgery of Axilla post-NAT^a^, n(%)			0.205
Sentinel lymph node biopsy	4 (3)	18 (9)	
Axillary lymph node dissection	114 (97)	191 (91.87)	

**a** NAT: neoadjuvant therapy, **b** HR: hormone receptor, **c** TILs: tumor-infiltrating lymphocytes, **d** M-H: moderate-high, e △ADC_0-2_%: change rate of ADC after two cycles of neoadjuvant therapy.

For patients receiving HP combined with chemotherapy (**Table 5**), tpCR was more common in those with HR negativity (*P* = 0.004), high Ki-67 levels (*P* = 0.003), M-H infiltrated TILs (*P* = 0.011), ΔADC_0-2_% > 36.2% (*P* < 0.001), HER2 3+ (*P* = 0.008), and the TCb regimen (*P* = 0.013). For patients receiving HPy plus chemotherapy (**Table 6**), tpCR was significantly associated with M-H infiltration of TILs (*P* = 0.008), ΔADC_0-2_% > 36.2% (*P* = 0.003), and HER2 3+ (*P* = 0.007).

**Table 5:** Comparison of clinicopathological characteristics between the pCR and non-pCR groups in patients receiving HP plus chemotherapy.

	Pathological response after NAT^a^		
Characteristics	non-pCR (n=87)	pCR (n=171)	*P* Value
Age, n(%)			0.359
≤50	38 (44)	85 (50)	
>50	49 (56)	86 (50)	
Menstrual Status, n(%)			0.335
Premenopause	48 (55)	105 (61)	
Postmenopause	39 (45)	66 (39)	
Pre-NAT^a^ Clinical T Stage, n(%)			0.364
cT1	9 (10)	29 (17)	
cT2	63 (73)	114 (67)	
cT3	6 (7)	16 (9)	
cT4	9 (10)	12(7)	
Pre-NAT^a^ Clinical N Stage, n(%)			0.063
Negative	4 (5)	20 (12)	
Positive	83 (95)	151 (88)	
Pre-NAT^a^ Clinical TNM Stage, n(%)			0.327
II	50 (57)	109 (64)	
III	37 (43)	62 (36)	
Pre-NAT^a^ Histopathological Type, n(%)			0.417
Invasive ductal carcinoma	72 (83)	148 (87)	
others	15 (17)	23 (13)	
Pre-NAT^a^ Histological Grade, n(%)			0.120
G1-2	70 (80)	150 (88)	
G3	17 (20)	21 (12)	
Pre-NAT^a^ HR^b^ State, n(%)			0.004
Negative	28 (32)	87 (49)	
Positive	59 (68)	84 (51)	
Pre-NAT^a^ HER2 Expression, n(%)			0.008
2 +	13 (15)	9 (5)	
3 +	74 (85)	162 (95)	
Pre-NAT^a^ ki-67 Expression, n(%)			0.003
≤ 30%	52 (60)	69 (40)	
> 30%	35 (40)	102 (60)	
Pre-NAT^a^ TILs^c^ Infiltration, n(%)			0.011
Low infiltrated	62 (71)	94 (55)	
M-H^d^ infiltrated	25 (29)	77 (45)	
ΔADC_0-2_%^e^, n(%)			<0.001
≤ 36.2%	63 (72)	85 (50)	
> 36.2%	24 (28)	86 (50)	
Chemotherapy Regimen for NAT^a^, n(%)			0.013
anthracyclines contained	11 (13)	23 (13)	
platinum contained	50 (57)	123 (72)	
monoalbumin-bound paclitaxel	26 (30)	25 (15)	
Surgery of Breast post-NAT^a^, n(%)			0.377
Mastectomy	12 (14)	31 (18)	
Breast-conserving surgery	75 (86)	140 (82)	
Surgery of Axilla post-NAT^a^, n(%)			0.113
Sentinel lymph node biopsy	3 (3)	15 (9)	
Axillary lymph node dissection	84 (97)	156 (91)	

**a** NAT: neoadjuvant therapy, **b** HR: hormone receptor, **c** TILs: tumor-infiltrating lymphocytes, **d** M-H: moderate-high, **e** △ADC_0-2_%: change rate of ADC after two cycles of neoadjuvant therapy.

**Table 6:** Comparison of clinicopathological characteristics between the pCR and non-pCR groups in patients receiving HPy plus chemotherapy.

	Pathological response after NAT^a^		
Characteristics	non-pCR (n=31)	pCR (n=38)	
Age, n (%)			0.614
≤50	12 (39)	17 (45)	
>50	19 (61)	21 (55)	
Menstrual Status, n (%)			0.726
Premenopause	15 (48)	20 (53)	
Postmenopause	16 (52)	18 (47)	
Pre-NAT^a^ Clinical T Stage, n (%)			0.752
cT1	5 (16)	4 (11)	
cT2	20 (65)	24 (63)	
cT3	4 (13)	5 (13)	
cT4	2 (6)	5 (13)	
Pre-NAT^a^ Clinical N Stage, n (%)			0.187
Negative	1 (3)	6 (16)	
Positive	30 (97)	32 (84)	
Pre-NAT^a^ Clinical TNM Stage, n (%)			0.328
II	23 (74)	24 (63)	
III	8 (26)	14 (37)	
Pre-NAT^a^ Histopathological Type, n (%)			0.138
Invasive ductal carcinoma	26 (84)	26 (68)	
others	5 (16)	12 (32)	
Pre-NAT^a^ Histological Grade, n (%)			0.776
G1-2	27 (87)	35 (92)	
G3	4 (13)	3 (8)	
Pre-NAT^a^ HR^b^ State, n (%)			0.104
Negative	8 (26)	17 (45)	
Positive	23 (74)	21 (55)	
Pre-NAT^a^ HER2 Expression, n (%)			0.007
2 +	7 (23)	0 (0)	
3 +	24 (77)	38 (100)	
Pre-NAT^a^ ki-67 Expression, n (%)			0.484
≤ 30%	14 (45)	14 (37)	
> 30%	17 (55)	24 (63)	
Pre-NAT^a^ TILs^c^ Infiltration, n (%)			0.008
Low infiltrated	27 (87)	22 (58)	
M-H^d^ infiltrated	4 (13)	16 (42)	
ΔADC_0-2_%^e^, n(%)			0.003
≤ 36.2%	24 (77)	16 (42)	
> 36.2%	7 (23)	22 (58)	
Chemotherapy Regimen for NAT^a^, n(%)			0.588
anthracyclines contained	16 (52)	20 (53)	
platinum contained	2 (6)	5 (13)	
monoalbumin-bound paclitaxel	13 (42)	13 (34)	
Surgery of Breast post-NAT^a^, n(%)			0.459
Mastectomy	23 (74)	31(82)	
Breast-conserving surgery	8 (26)	7 (18)	
Surgery of Axilla post-NAT^a^, n(%)			0.758
Sentinel lymph node biopsy	1 (3)	3 (8)	
Axillary lymph node dissection	30 (97)	35 (92)	

**a** NAT: neoadjuvant therapy, **b** HR: hormone receptor, **c** TILs: tumor-infiltrating lymphocytes, **d** M-H: moderate-high, **e** △ADC_0-2_%: change rate of ADC after two cycles of neoadjuvant therapy.

### Multivariate regression analyses of factors affecting tpCR

The multivariate analysis (**Table 7**) revealed that HR negativity (odds ratio [OR], 2.86; 95% CI: 1.64, 4.99; *P* < 0.001), HER2 3+ (OR, 4.63; 95% CI: 1.82, 11.79; *P* = 0.001), high Ki-67 index (OR, 2.52; 95% CI: 1.47, 4.32; *P* < 0.001), H-M infiltrated TILs (OR, 2.47; 95% CI: 1.38, 4.40; *P* = 0.002), and ΔADC_0-2_% > 36.2% (OR, 3.68; 95% CI: 2.10, 6.44; *P* < 0.001) were independent predictive indicators for tpCR.

**Table 7:** Multivariate binary logistic regression analysis of variables for their association with pCR after NAT^a^.

Characteristics	Odds ratio	95% CI	*P* Value
Intercept	0.14	0.03, 0.73	0.02
Pre-NAT^a^ Clinical Lymph Node Metastasis			
Negative	Reference		
Positive	0.42	0.14, 1.22	0.11
Pre-NAT^a^ HR^b^ State			
Positive	Reference		
Negative	2.86	1.64, 4.99	<0.001
Pre-NAT^a^ HER2 Expression			
2 +	Reference		
3 +	4.63	1.82, 11.79	0.001
Pre-NAT^a^ Ki-67 Expression			
≤ 30%	Reference		
> 30%	2.52	1.47, 4.32	<0.001
Pre-NAT^a^ TILs^c^ Infiltration			
Low infiltrated	Reference		
M-H^d^ infiltrated	2.47	1.38, 4.40	0.002
ΔADC_0-2_%^e^			
≤ 36.2%	Reference		
> 36.2%	3.68	2.10, 6.44	<0.001
Chemotherapy Regimen for NAT^a^			
anthracyclines contained	Reference		
platinum contained	1.85	0.96, 3.58	0.07
monoalbumin-bound paclitaxel	0.78	0.37, 1.64	0.51

**a** NAT: neoadjuvant therapy, **b** HR: hormone receptor, **c** TILs: tumor-infiltrating lymphocytes, **d** M-H: moderate-high, **e** △ADC_0-2_%: change rate of ADC after two cycles of neoadjuvant therapy.

### Nomogram development and validation

**Figure 4:**
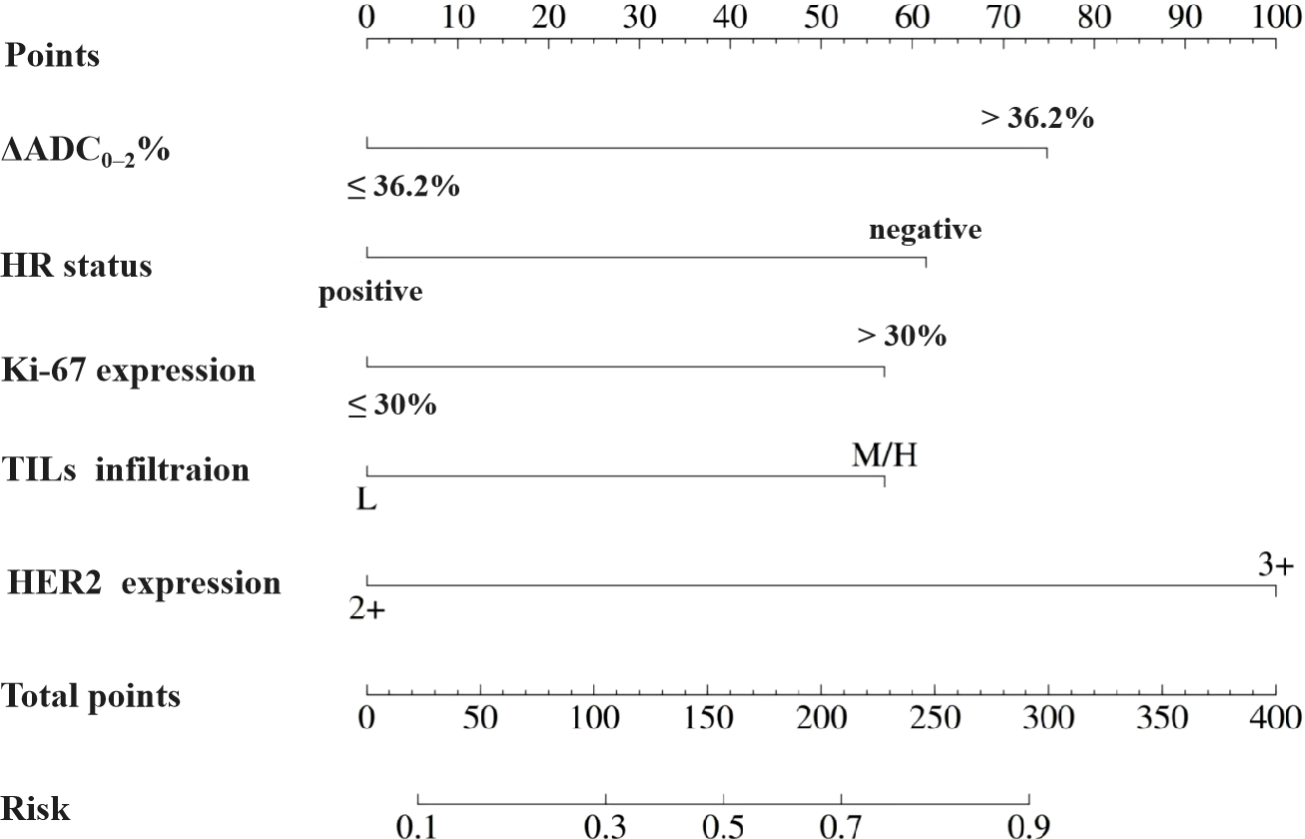
Nomogram for pCR prediction following neoadjuvant therapy in HER2-positive breast cancer.

**Figure 5:**
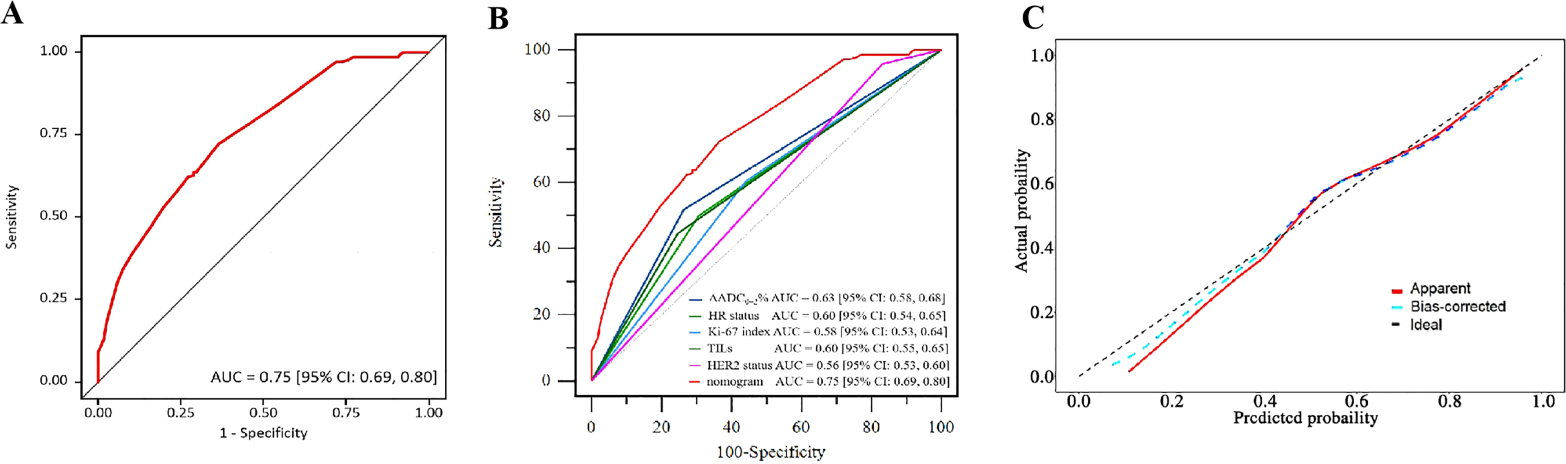
Validation of the nomogram. **(A)** ROC of the nomogram. **(B)** ROC of the variables and the nomogram. **(C)** Calibration plot of the nomogram.

**Figure 6:**
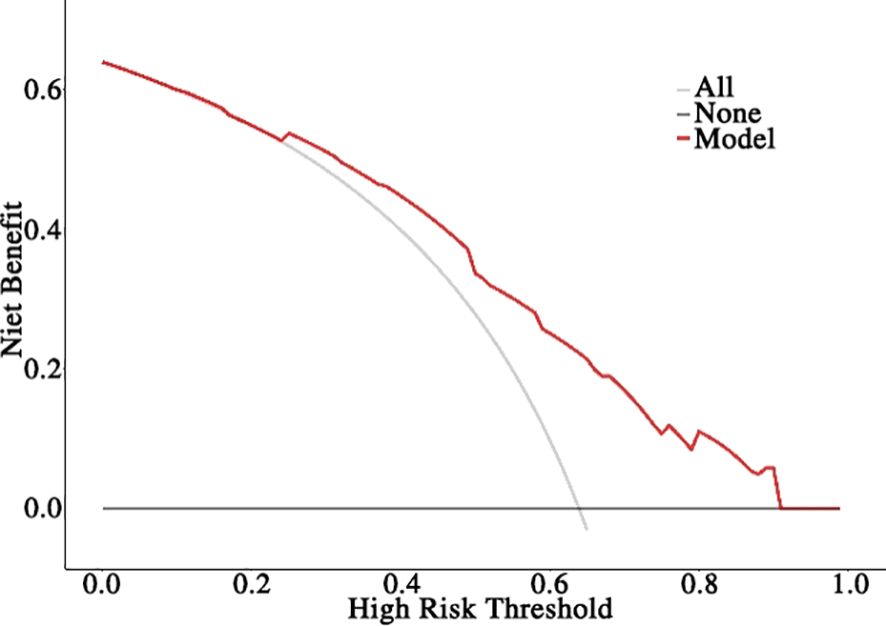
The decision curve analysis (DCA) for the nomogram.

Based on the aforementioned results, the nomogram was constructed using HR status, HER2 expression, Ki-67 index, TILs infiltration, and ΔADC_0-2_% as predictors. The corresponding score for each predictor was summed to generate a risk value that reflected the probability of achieving tpCR (**Figure 4**). The nomogram demonstrated good discrimination capability, as evidenced by ROC curve analysis (**Figure 5A**), with an AUC of 0.75 [95% CI: 0.69, 0.80] (*P* = 0.001). Internal validation of the nomogram model was performed using the bootstrap resampling method with 1,000 repetitions, which confirmed its high discriminative ability (AUC: 0.73). Furthermore, the nomogram was compared with individual variables (HR status, HER2 expression, Ki-67 index, TILs infiltration, and ΔADC_0-2_%), and it showed superior performance (**Figure 5B**). The calibration curve indicated good calibration performance of the nomogram (χ² = 3.14, df = 8, P = 0.925) (**Figure 5C**). DCA (**Figure 6**) further demonstrated the excellent clinical application value of the nomogram model.

## Discussion

This study retrospectively reviewed the pathological remission status of NAT for HER2-positive BC and compared the efficacy of HP and HPy targeted therapies combined with chemotherapy, yielding results similar to those of previous studies (27–30). Through univariate and multivariate regression analyses, HR status, HER2 expression, Ki-67 index, TILs infiltration, and ΔADC_0-2_% were included to develop a predictive model for tpCR. The model demonstrated excellent performance compared with individual variables and good clinical applicability. The variables included in the nomogram are common and readily available for clinical practice, facilitating its widespread application.

Based on published evidence, HP combined with chemotherapy is the preferred NAT regimen recommended by various guidelines (31, 32). Py, a novel oral irreversible tyrosine kinase inhibitor (TKI) targeting HER1, HER2, and HER4, was initially approved in China for the treatment of HER2-positive advanced or metastatic BC in 2018. By covalently binding to ATP at the intracellular kinase domains, Py inhibits the formation of homodimers and heterodimers as well as the auto-phosphorylation of the HER family. This action blocks the activation of downstream signaling pathways and inhibits the tumor cell cycle at the G1 phase, restricting tumor progression (33, 34). Due to the different mechanisms of action of H and Py, studies have explored their combination efficacy in early (6) and advanced HER2-positive BC (35). The results of the phase 3 PHERDA study indicated that HPy significantly improved the pCR rate compared to H monotherapy combined with chemotherapy (41.0% vs 22%, *P* < 0.0001) (6), thereby establishing the role of Py in NAT for HER2-positive BC. Currently, several studies have investigated the efficacy and safety of different chemotherapy regimens plus HPy (28–30, 36–41). Studies exploring TCb plus HPy demonstrated tpCR rates of 52%-73% (30, 36–38, 41). Other studies revealed that the tpCR rate for AC-T combined with HPy ranged from 63% to 73% (28, 29, 39, 41). Zhong et al. (40) reported that a T+H (weekly) plus Py regimen achieved a tpCR of 57.1%. A multicenter retrospective study involving 107 patients compared the efficacy of 4*T, 6*TCb/4*P (cisplatin), and 4*AC-4*T plus HP, suggesting that long-cycle taxane and platinum-containing regimens had higher tpCR and bpCR rates (42). In this study, the pCR rates for AC-T/TA, TCb, and T in combination with HPy (excluding patients without ADC values after 2 cycles of NAT) were 56% (20/36), 71% (5/7), and 50% (13/26), respectively, which were consistent with previous studies. However, due to the lack of large-scale, prospective, high-quality randomized controlled clinical trials (RCTs), the optimal chemotherapy regimen to be combined with HPy has not yet been determined. Considering patients’ age, general condition, comorbidities, and the prominent adverse reaction of diarrhea associated with Py, tailored therapy should be considered.

Although both HP and HPy are currently optional anti-HER2 targeted combinations for HER2-positive BC, there is no definitive conclusion on which regimen is superior due to the lack of prospective, randomized head-to-head comparisons between them. A retrospective study compared the efficacy of the two targeted therapies combined with TCb (41), suggesting that the pCR rates were comparable (TCb+HPy: 55.6%, TCb+HP: 56.6%). Further subgroup analyses confirmed that there was no difference in pCR rates between the two targeted combinations regardless of HR status and HER2 expression. A meta-analysis incorporating nine studies with a total of 1,745 patients also reached a similar conclusion (43). In our study, the efficacy of HP and HPy was evaluated. PSM was performed to account for confounding factors such as gender, age, and histological grade. After PSM, there was no statistically significant difference in pCR rates between the two regimens, indicating comparable efficacy. Since this was a retrospective study, AEs could not be fully collected, and the safety of the two combinations was not compared. Given that the prominent AE of Py is diarrhea, while the addition of P to H does not add extra AEs, the general condition of the patient, comorbidities, economic status, drug availability, and patient preference should be considered when selecting an appropriate combined regimen.

Previous studies have employed clinical and pathological parameters similar to those used in our study to construct predictive models. For instance, Yang et al. (44) utilized ER and PR expression, Ki-67 index, and HER2 status to build a predictive model for HER2-positive BC. The AUC was 0.73, and further validation demonstrated good discrimination and calibration. Similarly, Fujii T et al. (45) incorporated IHC biomarkers (ER, PR, and HER2 expression), clinical manifestation (inflammatory breast cancer [IBC] vs. non-IBC), and NAT regimen. However, this model was less discriminative (C-index: 0.69) and lacked determination of clinical usefulness. Compared to IHC biomarkers and NAT regimens, MRI image characteristics can more objectively and precisely reflect the nature and changes of lesions. Therefore, MRI parameters should be recommended for inclusion in predictive model development.

Several studies have utilized a wide variety of MRI parameters to construct models to forecast pCR following NAT. For example, Li et al. (46) developed a predictive model for HER2-positive BC that integrated radiomics based on contrast-enhanced MRI (CE-MRI), which showed good calibration, discrimination, and superior clinical usefulness. van der Voort A et al. (47) applied DWI combined with DCE-MRI but found no added value in identifying pCR for early HER2-positive BC. Kim SY et al. (48) introduced multiple indicators, including pre-NAT characteristics (tumor size, lesion type, rim enhancement, and peritumoral edema) and post-NAT characteristics (tumor size, lesion-to-background parenchymal signal enhancement ratio [SER]). Although the integration of various indices can enhance model performance, it involves a significant workload and is inconvenient for clinicians to apply in routine clinical practice.

The ADC value is commonly used to evaluate the response to NAT. Moreover, increases in ADC value during NAT have been shown to be more valuable than changes in tumor size or volume after treatment (49). Previous studies have investigated the association between ADC value and pCR, but the conclusions have been contradictory. For example, some studies (24, 50, 51) suggested that a low pretreatment ADC value is more likely to achieve pCR, while others (21, 47, 52, 53) did not identify a significant association between pCR and pretreatment ADC values. Two factors may contribute to these conflicting results: First, the lack of standardization of ADC values regarding scanner technology, equipment, and imaging sequences/protocols (54), which leads to diverse ADC values; Second, pretreatment ADC values vary across different molecular subtypes of BC (24). Therefore, the change rate of ADC values after NAT may be a preferable alternative.

Consequently, in this study, we utilized the change rate of ADC values during NAT. However, a key question remains: Which time point of the change rate should be used? Currently, there is no consensus on this issue (12, 21, 51, 55). Evidence suggests that the change rate of ADC value after two cycles of NAT is more indicative of pCR (9, 55). In this study, we also investigated the ADC value change rate following two cycles (ΔADC_0-2_%) of treatment and determined the optimal cutoff for pCR to be 36.2%. However, Lu et al. (12) demonstrated that only an ADC value change below 15% was related to pCR (OR = 9.865, 95% CI 1.024–95.021). The different cutoff values may result from disparate study cohorts, as treatment response varies with underlying molecular subtypes and tumor biology (21). This highlights the importance of constructing predictive models specific to various molecular subtypes. Additionally, our cutoff value of ΔADC_0-2_% is higher than that reported in a previous study (12), which may be attributed to the higher rate of pCR observed in our cohort.

This study has several limitations. Firstly, it was a retrospective, single-center study, which is susceptible to selection bias. Secondly, the limited sample size of patients receiving the HPy regimen may reduce the persuasiveness of the comparison between HPy and HP. Thirdly, given that the collection of AEs in retrospective studies may be incomplete, no summary or comparison of AEs was performed. Fourthly, external validation based on data from other institutions was not conducted; thus, verifying the performance of our nomogram through external validation is essential. And lastly, we only selected the rate of change in ADC values after two cycles of NAT, future work should analyze more time points.

## Conclusions

In conclusion, this study retrospectively reviewed the efficacy of NAT in patients with HER2-positive BC from a single center, comparing the pathological response of the combinations of HP or HPy with chemotherapy. A nomogram integrating the early change rate of ADC values and clinicopathological variables was developed to predict pCR, demonstrating good performance and clinical utility. Further head-to-head randomized clinical trials are needed to confirm the benefits and risks of HP and HPy plus chemotherapy. Additionally, external validation studies should be conducted to validate our nomogram model.

## Data Availability

The original contributions presented in the study are included in the article/supplementary material. Further inquiries can be directed to the corresponding author.
